# Viral antibody dynamics in a chiropteran host

**DOI:** 10.1111/1365-2656.12153

**Published:** 2013-11-13

**Authors:** Kate S. Baker, Richard Suu‐Ire, Jennifer Barr, David T. S. Hayman, Christopher C. Broder, Daniel L. Horton, Christopher Durrant, Pablo R. Murcia, Andrew A. Cunningham, James L. N. Wood

**Affiliations:** ^1^ Disease Dynamics Unit University of Cambridge Cambridge UK CB3 0ES; ^2^ Institute of Zoology Zoological Society of London London UK NW1 4RY; ^3^ Wildlife Division Forestries Commission Accra Ghana PO Box 239; ^4^ Australian Animal Health Laboratories Commonwealth Scientific and Industrial Research Organisation Geelong Vic Australia 3219; ^5^ Department of Biology Colorado State University Fort Collins CO USA 80523; ^6^ Department of Microbiology and Immunology Uniformed Services University of the Health Sciences Bethesda MD USA 20814‐4799; ^7^ Wildlife Zoonoses and Vector‐Borne Diseases Research Group Animal Health and Veterinary Laboratories Agency Surrey UK KT15 3NB; ^8^ College of Medical Veterinary and Life Sciences University of Glasgow Glasgow UK G12 8QQ; ^9^ Present address: Wellcome Trust Sanger Institute Hinxton Cambridge UK CB10 1SA

**Keywords:** Hendra virus, immune response, infection persistence, Luminex, maternal immunity, Nipah virus, paramyxoviruses, serology, zoonosis

## Abstract

1. Bats host many viruses that are significant for human and domestic animal health, but the dynamics of these infections in their natural reservoir hosts remain poorly elucidated.
2. In these, and other, systems, there is evidence that seasonal life‐cycle events drive infection dynamics, directly impacting the risk of exposure to spillover hosts. Understanding these dynamics improves our ability to predict zoonotic spillover from the reservoir hosts.
3. To this end, we followed henipavirus antibody levels of >100 individual *E. helvum* in a closed, captive, breeding population over a 30‐month period, using a powerful novel antibody quantitation method.
4. We demonstrate the presence of maternal antibodies in this system and accurately determine their longevity. We also present evidence of population‐level persistence of viral infection and demonstrate periods of increased horizontal virus transmission associated with the pregnancy/lactation period.
5. The novel findings of infection persistence and the effect of pregnancy on viral transmission, as well as an accurate quantitation of chiropteran maternal antiviral antibody half‐life, provide fundamental baseline data for the continued study of viral infections in these important reservoir hosts.

## Graphical abstract

This is the first study of henipaviral infection dynamics in a fully closed population of the natural reservoir hosts. The authors detect population‐level infection persistence as well as increased periods of viral transmission related to life‐cycle events. These data help us to understand the seasonality of recurrent zoonotic spillovers of viruses from bat populations.

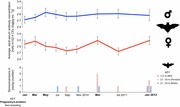

## Introduction

There are approximately 1200 bat species in the order *Chiroptera,* and these collectively act as reservoir hosts for a number of important viral zoonoses (Calisher *et al*. 2006; Luis *et al*. 2013). Bats are the natural host for lyssaviruses and are also the primary reservoirs for filoviruses, henipaviruses and SARS‐like coronaviruses (Halpin *et al*. 2000; Badrane & Tordo 2001; Li *et al*. 2005; Towner *et al*. 2009). The emergence of viral zoonoses from bats often has drastic consequences, such as the >150 human deaths associated with Nipah virus (NiV) emergence in Malaysia in 1999 (Chua *et al*. 2000). The trigger for initial emergences and drivers of exceptional increases in spillover frequency, such as the dramatic increase in Hendra virus spillover events in 2011 (Field *et al*. 2012), are often unknown and difficult to determine with so few events. However, bat‐derived viral zoonoses that do cause recurrent spillover events (such as henipaviruses and Marburg virus) often have a seasonal pattern (Luby *et al*. 2009; McFarlane, Becker & Field 2011). It is possible that this is related to seasonal changes in contact rates between reservoir and spillover hosts (e.g. animal stocking densities and caving tourism), but this has not been shown for Hendra or Marburg viruses (McFarlane, Becker & Field 2011; Amman *et al*. 2012). In the latter case, seasonal changes in zoonotic Marburg virus infections are suggested to be directly related to altered viral excretion from reservoir hosts (Amman *et al*. 2012). To continue exploration of these possibilities with the aim of anticipating spillover events, we need to understand the factors driving viral infection dynamics in bats.

Studies to date on the viral infection dynamics of various viruses in bats have revealed that some aspects may be near‐universal, regardless of the virus–host system examined. For example, the infection status of bat populations has been shown to be affected by season for henipaviruses, lyssaviruses, coronaviruses and filoviruses (Breed *et al*. 2011; George *et al*. 2011; Amman *et al*. 2012; Drexler *et al*. 2012a). This is likely because, as for many other wildlife classes (Hosseini, Dhondt & Dobson 2004; Altizer *et al*. 2006), bat life cycles are highly seasonal, with tightly synchronized breeding, hibernacula formation and migrations that will drive epidemiology by controlling key factors such as contact rates and the introduction of susceptible animals (George *et al*. 2011; Drexler *et al*. 2012a). This is supported by studies which show that the infection and immunological status of individual bats are affected by age, breeding phase and nutritional stress for some viruses (Gloza‐Rausch *et al*. 2008; Plowright *et al*. 2008; Breed *et al*. 2011). Further aspects of individual virus infection, such as maternal antiviral antibodies and persistent infection of individuals, likely affect infection dynamics in bats (Plowright *et al*. 2011) as in other systems (Kallio *et al*. 2006, 2010). The commonalities observed across different virus and bat–host relationships, and the challenge of fully characterizing the infection dynamics of a single pathogen–host system, make selecting a disease model for detailed study appropriate.

Hendra and Nipah viruses (in the genus *Henipavirus*) represent an important and useful model for the study of viral infection dynamics in bats. These paramyxoviruses cause fatal respiratory and encephalitic disease in a wide range of susceptible spillover hosts (including humans), while bats are apparently clinically unaffected by infection (Murray *et al*. 1995; Chua *et al*. 2000; Halpin *et al*. 2000). Consequently, henipaviruses must be worked with in highly rated biosecure (PC4) laboratories. These viruses cause recurrent disease outbreaks in Bangladesh and Australia (Luby *et al*. 2009; McFarlane, Becker & Field 2011; Field *et al*. 2012; Lo *et al*. 2012), and are of potential concern to a much greater geographical area with evidence of infection being near‐universally distributed throughout reservoir hosts in the Old World (Reynes *et al*. 2005; Wacharapluesadee *et al*. 2005; Sendow *et al*. 2006; Iehle *et al*. 2007; Hayman *et al*. 2008; Li *et al*. 2008). In addition to their continued clinical relevance, they are harboured by bats in the family *Pteropodidae*: reservoir hosts that are large enough to tolerate repeated serum sampling at practicable volumes and, as frugivores, relatively easy to maintain in captivity.

Observational field studies provide some information on henipaviral infection dynamics in bats, but such studies, owing to their nature, have limited scope. Demographic analysis of data from serial cross‐sectional sampling suggest that antihenipavirus maternal antibodies exist, that the viruses are horizontally transmitted among populations and that pregnancy and lactation may affect serological status (Plowright *et al*. 2008; Breed *et al*. 2011). More recently, a study into a very small, closed island population of bats showed population‐level persistence of henipaviral infection (Peel *et al*. 2012). This is inconsistent with traditional paramyxovirus epidemiology theory, where large populations are considered to be necessary for infection maintenance (Pomeroy, Bjornstad & Holmes 2008; Plowright *et al*. 2011). In order to investigate these inferences from field observations and accurately measure infection parameters for practical and theoretical studies, as well as to determine possible mechanisms of persistence, it is necessary to reliably, repeatedly and comprehensively sample individuals in a closed study population.

The resampling rates and closure of a study population to new infection required to demonstrate these effects are difficult to achieve in the wild. Old World fruit bat populations are often migratory and/or nomadic and extremely numerous (up to millions of individuals), making recapture unlikely (Hayman *et al*. 2012b). Opportunistic sampling through wildlife rehabilitation centres and zoological enclosures is complicated by the rolling entry of bats of unknown infection status (Field 2005; Rahman *et al*. 2010; Sohayati *et al*. 2011), and experimental infections are often of test subjects with unknown historic or current infection status (and the added complications of working with PC4‐classified agents on often‐protected test species) (Williamson *et al*. 1998; Middleton *et al*. 2007; Halpin *et al*. 2011). For these reasons, a purpose‐built facility of newly captive, breeding African straw‐coloured fruit bats (*Eidolon helvum*), naturally infected with, as yet unknown, henipavirus(es) was used to observe henipavirus antibody dynamics in chiropteran hosts over a 30‐month period.

## Ethics declaration

This study was approved by the Zoological Society of London's Ethics Committee.

## Materials and methods

### Sequentially sampled sera

Serum samples (*n* = 634) collected longitudinally from individually identified *E. helvum* maintained in captivity (*n* = 111) were analysed in this study.

### The captive population

**Figure 1:**
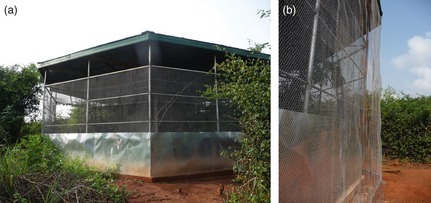
Captive facility for closed bat colony. (a) Hexagonal structure 27·5 m in diameter and 3·5 m in height. Walls and flat ceiling composed of steel mesh with a hole size of 25 mm, topped with a capped solid tin roof. Tin sheet cladding (1 m high) surrounded the base to prevent entry of terrestrial animals. (b) Modification added from eave of cap roof in January 2010 (when fully populated) to prevent contact with volant animals.

Bats were maintained in a large cage (closed to public view) in the grounds of Accra Zoological Gardens in Achimota Forest Reserve, Accra, Ghana, *c*. 6 km from where they were captured (Fig. 1a). The facility prevented contact with other animals through ground‐level cladding, second‐layering of mesh walls and ceiling (Fig. 1b) and a solid roof. Between July 2009 and January 2010, the facility was populated by three cohorts (1–3, Table 1) totalling 77 wild *E. helvum* of admixed age and sex. These bats were captured from a large seasonal population in the grounds of 37 Military hospital in Accra, Ghana (Hayman *et al*. 2012b). This wild population is known to be infected with henipaviruses (Hayman *et al*. 2008). Continued identification of individuals was ensured by subcutaneous passive integrated transponder (PIT) tag implantation in each bat and also the use of ball‐bearing necklaces carrying marked stainless steel butt‐end rings (Bat ID, Table S1) on fully‐grown bats. The sex and age at entry of each bat were recorded according to the following criteria: fully‐grown bats with secondary sexual characteristics (descended testes or previously suckled nipples) were deemed ≥24 months of age and termed adults (A); bats not fully‐grown were assumed born in the previous breeding season (i.e. <12 months old) and termed juveniles (JUV). Finally, bats fully‐grown but with no secondary sexual characteristics were classified as sexually immature (SIM) and as having been born in the penultimate breeding season (i.e. between 12 and 24 months old). Two further entry cohorts totalling 33 *E. helvum* were born in the facility and termed ‘born in captivity’ (BIC). Cohort 4 (born in 2010) resulted from wild matings, and Cohort 5 (born in 2011) resulted from captive matings (Table S1). The age of bats < 24 months old was inferred from a presumed birth date of April 1st each year. This date was based on observations in the captive population and the local wild population (Hayman *et al*. 2012b). Bats exited the study through mortality on known dates (*n* = 12) or presumed dates where they were unaccounted for over a period of ≥3 sampling intervals (*n* = 11, Table S1). This time interval was selected as bats missing for only two events had eventually been resampled.

**Table 1:** Sampling and entry dates of bat cohorts, and their composition with respect to age and gender. Age group abbreviations are sexually immature (SIM), juvenile (JUV) and born in captivity (BIC). For non‐adult age groups, approximate age in months (m) of bats at entry is shown in parentheses. Gender abbreviations are male (M), female (F) and not determined (ND)

Date	Sampling Intervals		Number of bats entering (by age group and gender)										
	Time (days) since		Cohort number	Age	Adult		SIM (months)		JUV (months)		BIC		
	Study start	Last sampling		Gender	M	F	M	F	M	F	M	F	ND
27th July 09	0	0	1		11		1 (15m)						
5th November 09	101	101	2		5	3		3 (19m)	2 (7m)				
28th January 10	185	84	3		12	29	3 (21m)	3 (21m)	2 (9m)	4 (9m)			
6th March 10	222	37											
1st April 10	246	No sampling	4								4	7	
21st May 10	298	76	Born in										
14th July 10	352	54	2010										
23rd September 10	423	71											
5th November 10	466	43											
4th March 11	585	119											
1st April 11	611	No sampling	5								3	8	11
13th July 11	716	131	Born in										
17th January 12	904	188	2011										
			Total	(111)	28	32	4	6	4	4	7	15	11

### Population sampling and determination of maternity

Serum samples were taken from the bats at 11 time points over a 30‐month period (Table 1). Pregnancy status (determined by palpation) at sampling was also recorded (Table S1). Maternal identity of pups was noted as the dam suckling them (or to which they were attached) at first capture. In total, 13 dam‐pup pairs were identified across two birthing seasons (Table S1). Bats were captured by being corralled into one‐quarter of the facility (using a curtain system) before individual capture by hand and temporary holding in cloth bags. Following throat swabbing and serum sampling (as previously described (Hayman *et al*. 2012a; Peel *et al*. 2012)), bats were released into the remainder of the enclosure. Due to escape of some bats from the subenclosure to the main area during capture, sampling of the population was sometimes incomplete (see Tables 2, 3 S1 and S2).

### Serological testing: antibody detection

A previously described assay based on Luminex technology was used to detect antihenipavirus antibodies (Bossart *et al*. 2007). Briefly, 30 μg of a soluble dimeric form of the NiV glycoprotein (NiVsG) (Bossart *et al*. 2005) was conjugated to 1·25 × 10^6^ polystyrene microspheres (Bio‐Rad, Hercules, CA, USA), which then acted as the testing surface for antibody capture. Conjugated beads were blocked in 2% [w/v] skimmed milk powder (Premier International, Hertfordshire, UK) before incubation with diluted sera (all bat sera were tested at a dilution of 1:50). Beads were then incubated with 2 μg mL^−1^ biotinylated protein A (Pierce, Rockford, IL, USA) before incubation with 1 μg mL^−1^ streptavidin‐conjugated R‐phycoerythrin (Qiagen Venlo, Limburg, Netherlands). The NiVsG Median Fluorescence Intensity (MFI) of ≥100 beads was reported for each sample. Thus, the results of this assay are on a continuous scale (in contrast to SNT testing intervals). All field sera were tested in duplicate, with temporally sequential sera from an individual being tested on the same plate. For the confirmation of the IgG isotype antibody in neonatal sera, the assay was performed as above, but with biotinylated protein A being substituted with goat anti‐bat‐IgG antibody (Bethyl laboratories, 1 μg mL^−1^) followed by biotinylated rabbit anti‐goat‐IgG (Bethyl laboratories, Montgomery, TX, USA, 1 μg mL^−1^). All serum samples were heat‐treated at 56 °C for 30 min prior to serological assay.

### Serological testing: antibody quantitation

A novel quantitation method was used to infer changes in henipavirus antibody concentration over time. Changes in NiVsG MFI were interpreted against a titration of a potently neutralizing antihenipavirus monoclonal antibody (mAb) m102·4 (Zhu *et al*. 2008). The specific batch of antibody (Lot: 20110328, NCRIS Biologics facility, 9·2 mg mL^−1^) had been shown to neutralize Hendra virus to a dilution of 1:30 000 (or 5·5 log[pg mL^−1^]) (Klein, Pallister, personal communication). The antibody was diluted in a 7‐point, 10‐fold dilution series (from 1:100 through to 1:100 000 000) previously shown to be effective for generating titration curves analogous to those achieved using more extensive dilution series (Baker, unpublished results). This standard titration was included in every run of the assay in which bat sera were tested.

The MFI replicates (*n* = 8) for each concentration of the mAb m102·4 standard were averaged and used to logistically fit a curve using the nonlinear least‐squares regression model within the R statistical package (R‐team 2006). The curve was logistically fitted using the following four parameters characteristic of immunoassay: slope, inflection point, maximum asymptote and minimum asymptote, which was constrained to ≥0 (Healy 1972; Grotjan & Keel 1996; Motulsky & Christopoulos 2004).

### Seroprevalence analyses

Where required, bats were classified as either seropositive or seronegative according to the serum antibody concentration; with results ≥2 mAb m102·4 concentration equivalents (CEs) (i.e. ≥100 pg mL^−1^) being classed as seropositive and results < 2 mAb m102·4 CEs being classed as seronegative. This level correlates with a cut‐off appropriate for the prediction of exposure in this species (Peel *et al*. 2013). Seroprevalences are shown as the proportion of the group that was seropositive, and binomial 95% confidence intervals were calculated using the Wilson method in R (Wilson 1927). Chi‐squared or Fisher's exact tests were used when comparing seroprevalence between demographic groups.

### Analysis of maternal antibody (matAb) waning

Antibodies in seropositive BIC bats at first sampling (≤3 months of age) were deemed to be maternal antibodies (for reasons outlined in the results and discussion). Least‐squares linear regressions were used to determine matAb half‐lives in individual bats. The overall waning rate of matAbs was determined for all data using a mixed‐effects linear regression model in the lme4 package, regressing time after birth (days) against serum antibody concentration (mAb m102·4 CEs), with individual bats incorporated as a random intercepts component (R‐team 2006).

### Seroconversions

Seroconversions were defined as a ≥ fourfold increase in antibody concentration in sequential samples (Thrusfield 2005). Notably, on the logarithmic scale used here, a fourfold increase in antibody concentration is equivalent to an increase in mAb m102·4 CEs of ≥0·6.

### Determination of the effect of breeding in adults

The effect of season (relative to pregnancy/lactation) on antibody concentration was examined in adult bats. Sampling events were classified as either occurring at the time of pregnancy/lactation in the population (sampling events in January – May inclusive, Table S1), or in a non‐breeding phase (sampling events outside of this time period). The effect of this phase on antibody concentrations was then examined using a mixed‐effects linear regression model using the lme4 package, regressing breeding phase against antibody concentration, with bats as a random intercept component and males and females being analysed separately (R‐team 2006). For model prediction, data points falling below the minimum asymptote of interpolatable antibody concentration (i.e. < 2 mAb m102·4 CEs) were conservatively considered as equal to 2 mAb m102·4 CEs, with models being reconfirmed when these values were considered equal to zero or omitted.

## Results

### Determination of changes in antibody concentration

**Figure 2:**
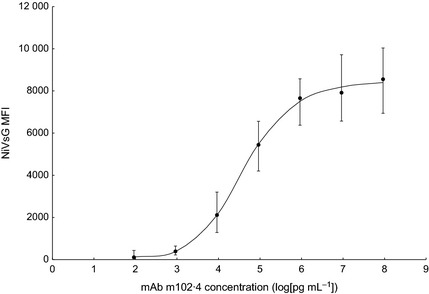
Relationship of NiVsG Median Fluorescence Intensity (MFI) with mAb m102·4 antibody concentration. The average NiVsG MFI of eight replicates for seven concentrations of mAb m102·4 are markers, with error bars showing the range of values obtained. The line is logistically fit to the averages using four parameters.

Antibodies that bound a soluble form of the NiV glycoprotein (NiVsG) were detected using a fluorescence‐based assay, which returns a continuous variable (Median Fluorescence Intensity or MFI). This variable correlates nonlinearly with serum antibody concentration, so the correlation was determined empirically. Titration of a potently neutralizing antihenipavirus antibody (mAb m102·4) was used to ascertain the change in MFI relative to serum antibody concentration. Eight replicates of the titration showed that MFI, and the variation in MFI, increased with antibody concentration (Fig. 2). The relationship was determined to be as follows: a curve was logistically fitted to the average of titration replicates using the four parameters: slope, inflection point, and maximum and minimum asymptotes which had the values: −7·724 mAb m102·4 (log[pg mL^−1^]), 4·61 mAb m102·4 (log[pg mL^−1^]), and 8521 and 112 MFI, respectively (Fig. 2).

The logistically fitted curve was then used to calculate antibody concentration from bat sera MFIs. Duplicate replicates of bat sera MFIs correlated well (*R* ^2^ = 0·93, not shown), so the average of replicates was used for calculations. Based on the relationship between MFI and mAb m102·4 concentration, sample antibody concentrations were given the unit mAb m102·4 concentration equivalents (CEs), with a value equivalent to the mAb m102·4 concentration which returned the same MFI as the sample. For samples with MFIs below the minimum asymptote of the curve, the antibody concentration was recorded as <2 mAb m102·4 CEs. The highest antibody concentration found in a bat serum sample was 4·3 mAb m102·4 CEs. The antibody concentrations for every sample in this study are presented in Table S1.

### Overall seroprevalence against henipaviruses

**Figure 3:**
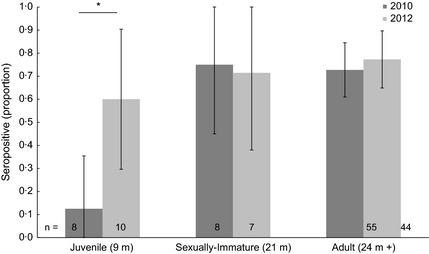
Seroprevalence of captive bat age groups at start (January 2010) and end (January 2012) of study. The sample size for each group is overlaid on columns, and error bars represent 95% confidence intervals of the proportion. Significant differences in seroprevalences are shown by an asterisk.

Seroprevalence of antihenipavirus antibodies in the population was evaluated at the start and end of the study to facilitate comparison with previous studies; contextualize observed changes in individual antibody fluctuations and to provide insight on the consequences of study design on population‐level infection. With respect to prior exposure, the study was considered to have started on 28th January 2010, when the final wild‐caught population cohort was added (Cohort 3, Table 1) and the facility was modified to prevent direct or indirect contact with wild bats (and hence infection, Fig. 1b). Age‐stratified seroprevalence at this time showed that approximately 15% of juvenile bats had detectable antihenipavirus antibodies, compared with ~70% of older (≥ 21 months) bats (Tables, Fig. 3), amounting to a weak positive association of serostatus with age (Fisher's exact test: *P* = 0·13). Comparatively, at the final time point of the study (17th January 2012), juvenile seroprevalence was significantly (χ^2^ test: *P* < 0·05) higher, at ~60%, whereas seroprevalences in older groups were equivalent (Fig. 3). At each time point, there was no significant difference in the seroprevalences between the sexes (χ^2^ tests: 0·07 and 1·40 for 2010 and 2012, respectively).

### Presence, waning and role of maternal antibodies

**Figure 4:**
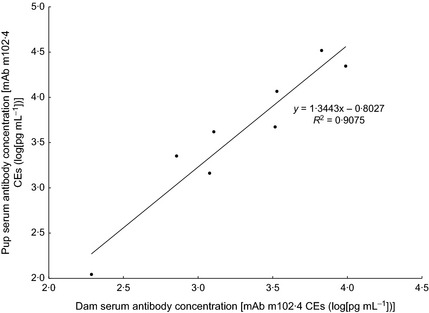
Correlation between serum antibody concentrations in seropositive dam‐pup pairs. A regression line with the equation and residual sum of squares is shown.

Laboratory analysis of neonatal sera and analysis of dam‐pup pairs demonstrated the presence of maternal antibodies (matAb). There was an association of serostatus and strong correlation of antibody concentrations in 13 known dam‐pup pairs. Nine seropositive pups were born to one seronegative and eight seropositive dams, and four seronegative pups were born to one seropositive and three seronegative dams (OR = 24, χ^2^ test: *P* < 0·05, Table S1). Furthermore, for the eight dual‐positive dam‐pup pairs, antibody concentrations were tightly correlated (*R* ^2^ = 0·90) with pups having slightly higher antibody concentration than their dams (Fig. 4). The neonate serum samples (first samplings at ≤3 months of age, *n* = 34) were tested using an anti‐bat‐IgG‐specific conjugate as well as the less‐discerning protein A conjugate used across the study. The tight correlation of fluorescence outputs generated by these two antibody conjugates (*R* ^2^ = 0·84, Fig. S1) demonstrated that observed reactivity was due to the presence of antihenipavirus antibodies of the IgG isotype, typical of matAb. Unfortunately, anti‐bat IgM conjugates are not commercially available.

The concentration of neonatal matAb universally declined in resampled pups (*n* = 14). Using sequential data from these individuals, the rate of matAb decay was estimated. Linear regressions on samples from individuals (typically calculated from two data points, details in Fig. S2) revealed a range of matAb half‐lives (between 40 and 97 days) with no clear relationship to extrapolated antibody concentrations at birth (Fig. S2). Mixed‐effects linear regression (which allowed for random intercepts, incorporating data from all individuals) estimated the matAb half‐life as 61 days (95%CI 56–66 days, Fig. S2). Typically, matAb was undetectable 4–12 months after birth.

**Figure 5:**
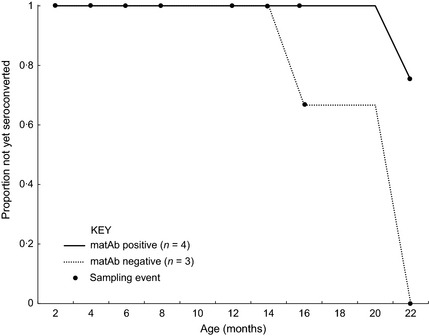
Age to seroconversion for bats born in captivity and tracked to adulthood. Proportion of bats that have not yet seroconverted is shown grouped by matAb status at first sampling.

To aid later discussion of the two pups born with different serostatus to their dams, a succinct note on their serological results over the time course of the study is as follows. The seropositive pup born to a seronegative dam in April 2010 (BatID: B153) had the lowest antibody concentration at birth of all the seropositive pups (Table 2, Fig. S2). Following decline in the concentration after birth (leaving it seronegative by 6 months of age), its subsequent seroconversion by 22 months of age made it the only seropositive‐born neonate to seroconvert over the course of the study (continued below, Table 2, Fig. 5). Meanwhile, the seronegative pup (BatID: 9186) born in April 2011 was born to a dam with an antibody concentration of 3·1 mAb m102·4CEs (BatID/ A144). This seronegative pup was the only one (of three born in 2011) to have seroconverted by their subsequent (and only) resampling event aged 10 months (Table 2), making it the youngest pup to seroconvert in the study (see below).

**Table 2:** Serum antibody concentrations at different sampling intervals for bats born in captivity in 2010 and 2011 with repeat sampling data. Grey shading denotes when a bat had exited the study and empty sites where the bat was not sampled. Sampling events where seroconversion has occurred relative to the previous sample are highlighted in bold

Date	Antihenipavirus antibody in mAb m102·4CEs (log[pg mL^−1^]) by sampling date										
		May‐10	July‐10	September‐10	November‐10	March‐11	July‐11	January‐12		July‐11	January‐12
Entry cohort		4 (Born in 2010)								5 (Born in 2011)	
Bat age	Months	2	4	6	8	12	16	22		4	10
	Days	51	105	176	219	338	469	657		104	292
BatID									BatID		
B188		<2	<2	<2	<2	<2	**3**		9186	<2	**2·9**
B111		<2		<2	<2	<2^a^			7034	<2	<2
B157^a^		<2	<2	<2	<2	<2		**2·7**	A175	<2	<2
B132		4·3	3·7	3·5	3	2·5	<2	<2	3940	4·6	3·7
B106^a^		4·1	3·4^a^		2·8			2·7	6544	4·4	3·9
B147		3·7	3·3^a^						A001	3·8	3·1
B150		3·4		<2	<2	<2		<2	7158	3·6	2·8
B120		3·2	2·9	<2	<2	<2	<2	2·2	A081	3·5	2·6
B153		2·8	2·5	<2	<2	<2	<2	**3·1**	A0004	3·4	2·4
BJ1			<2	<2	<2	<2	<2	**3**	3428	3·1	<2
									A075	2·2	<2

Excluded from calculations in Fig. 5 due to incomplete observations.

a Other BatIDs shown in Table S1 (i.e. band ID here was replaced and identification was by PIT‐tag).

### Seroconversions in subadult bats

This study afforded opportunities to observe seroconversions in three groups of subadult (<24 months) bats, and the relationships of each with the presence of matAb and other life‐cycle events were examined. The first was those bats born in captivity in 2010, which were sampled from 2 to 22 months of age (*n* = 7, Table 2). Four of these seven bats seroconverted between 16 and 22 months of age, with matAb‐negative bats seroconverting younger and to a greater proportion than matAb‐positive bats (Fig. 5, Table 2). Secondly, bats that entered the study in January 2010 as 9‐month old, wild‐captured juveniles (observed to 34 months of age) were also observed to seroconvert. Of the eight bats that entered the study as juveniles, six seroconverted between 16 and 24 months of age, with a further one seroconverting at 28–34 months of age at the end of the study (Table 3). One female wild‐caught juvenile (BatID: A098) seroconverted at 18 months of age, and then again at 24 months of age (Table 3), the latter event being timed with a broader trend observed in adult females (more below). Notably, the only wild‐caught juvenile that did not seroconvert (BatID: A112) was the only one with detectable (possibly maternal) antibodies on entry (Table 3). The third opportunity to observe seroconversions in young bats was the 10 month period following the birth of the 2011 cohort (Table 2). Thus, including the seronegative pup born to a seropositive dam described in the last section, a total of 12 seroconversions in subadult bats were observed throughout the study period. These seroconversions were concentrated around two time points (March 2011 and January 2012, Fig. 6).

**Table 3:** Serum antibody concentrations at different sampling intervals for bats that entered the study as young (either juvenile (JUV) or sexually immature (SIM) bats). Grey shading denotes when a bat had exited the study and empty sites where the bat was not sampled. Sampling events where seroconversion has occurred relative to the previous samples are highlighted in bold

Date		Antihenipavirus antibody in mAb m102·4CEs (log[pg mL^−1^]) by sampling date										
		July‐09	November‐09	January‐10	March‐10	May‐10	July‐10	September‐10	November‐10	March‐11	July‐11	January‐12
JUV (age in months)	Bat ID		8	10	12	14	16	18	20	24	28	34
	A112		3·4	3·6	<2	<2	<2		<2		<2	<2
	A101		<2	<2	<2	<2	**2·7**	3	3		3·1	
	A166^a^			<2	<2	<2	<2	<2	<2	**3·8**		
	A152			<2	<2	<2	<2	<2	<2	**4·5**	3·9	4·4
	A130			<2	<2	<2	<2	<2	2·5	**3·5**	3·7	
	A115			<2	<2	<2	<2	<2	**3·1**		**4·2**	4·4
	A099			<2	<2	<2		<2	<2	<2	<2	**4·4**
	A098			<2	<2	<2		**2·9**	3·1	**3·9**	3·8	4·1
SIM (age in months)	Bat ID	16	20	22	24	26	28	30	32	36	40	46
	A196	2·9	3·5	3·5		3·2	3·3	3·2	3·5	3·4	3·4	3·6
	A109		3		3·2	3·3	3·1	3	3			
	A108		4·4	4·5	4·7	4·3	4·5	4·7				
	A148			4·7	4·6	4·1	4·3	4·3			3·8	4·5
	A144			2·8	<2	<2	<2	<2	<2	**3·3**	3·1	3·2
	A134			<2	<2			<2	<2	<2	<2	<2
	A126			2·6	2·5	2·9		3	3	3	2·8	2·9
	A122			3·2	3	**3·7**	3·5	3·3	3·5	3·4	3·4	
	A097			<2	<2	<2	<2	<2	<2	**2·9**	3·7	3·8

a Other BatIDs shown in Table S1 (i.e. band ID here was replaced and identification was by PIT‐tag).

**Figure 6:**
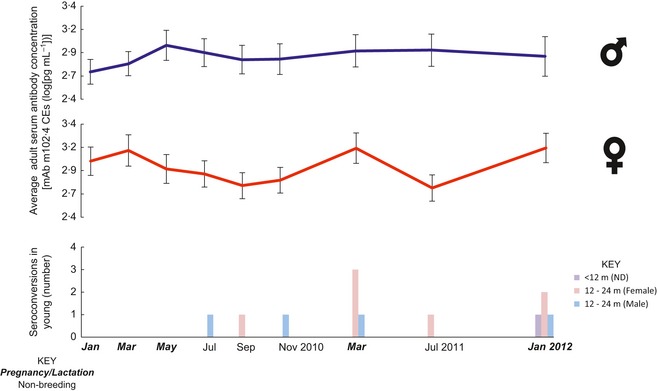
Fluctuations in antibody concentration over time in age and sex groups. The mean of antibody concentrations for all adult bats by sex is shown in the top graphs with error bars of the standard error overlaying sampling dates. The lowest graph depicts the timing and number of seroconversions in sex groups of subadult bats. ND is not determined. The axis shows the abbreviated sampling dates formatted by breeding phase.

### Seroconversions in adult bats

The large majority (~75%) of seroconversions in adult (>24 months) bats occurred in females in a synchronized fashion, while only three seroconversion events were detected in adult male bats. Two of the latter occurred in a single individual (BatID: A192) that entered the facility in July 2009 were seropositive throughout the entire study (between 2·4 and 4 mAb m102·4CEs) and which seroconverted in May 2010 and November 2010 (Table S2). The remaining adult male seroconversion occurred in a bat that entered the study with detectable antibodies (3·2 mAb m102·4CEs) at 22 months of age and seroconverted 4 months later (to 3·7 mAb m102·4CEs, also in May 2010) before equalizing for the remainder of the study (Table 3). Although infrequent, seroconversions in adult males were the first seroconversions observed, some 4 months into the study.

Finally, eleven seroconversions were observed in adult females: one detected in November 2011, seven in March 2011 and three in January 2012 (Table S2). The seroconversions in March 2011 and January 2012 occurred during late pregnancy/lactation and were each contemporaneous with the seroconversion of four young bats in the study (Fig. 6). Further investigation of antibody concentration with season showed further non‐seroconverting (i.e. <fourfold) temporal increases in individual antibody concentration associated with pregnancy/lactation (Table S2, Fig. S3); a trend which was lacking in adult males (Figs 6, S3). To quantify this effect of breeding, a random intercepts model was fitted to determine the impact of breeding phase by sex in adult bats. This model demonstrated a relative increase (1·9‐fold, 95%CI 1·6–2·2) in antibody concentration in individual adult females during the pregnancy/lactation season (*P* < 0·01, Table 4). In contrast, adult male antibody levels were unaffected by season (equivalent figures are a onefold change, 95% CI 0·9–1·2, *P* > 0·05, Table 4). In fact, the pregnancy/lactation phase accounted for most of the temporal change in adult antibody concentration, with the remaining variation in each model being primarily attributable to the variation in starting antibody concentration of individual bats (not explored here) and residual variance being only ~15% of this figure.

**Table 4:** Mixed‐effect model parameters for regression of reproduction effect on adult bat antibody concentration by sex

Linear mixed‐effect model parameters for antibody concentration [mAb m102·4 CEs (log [pg mL^−1^])] in adult bats			
	Parameter	Sex	
		Female	Male
Number of	Observations	213	191
	Bats	28	24
Fixed effects (95% CI)	Intercept	3·04 (2·77, 3·31)	2·86 (2·58, 3·13)
	Non‐Breeding	−0·28 (0·21, 0·35)	0·00 (−0·07, 0·07)
Random effects (SD)	Individual (Bat)	0·51 (0·71)	0·44 (0·67)
	Residual	0·06 (0·25)	0·07 (0·26)

## Discussion

Here, we used the changes in antihenipavirus antibody concentration of a newly captive breeding population of *E. helvum* to investigate fundamental aspects of viral infection dynamics in a chiropteran host.

Before discussing the serological results of this study, it is worth considering what is (and is not) known about the virus in question. No henipavirus has yet been isolated from Africa, so the preference to work within a fully characterized host–pathogen system could not be fulfilled here. However, despite the complex relationship between bats and paramyxoviruses, some inferences about the virus (or viruses) likely responsible for inducing the production of these antibodies can be made. Fragments of many henipa‐like viruses have been detected in this bat species (Drexler *et al*. 2009, 2012b; Baker *et al*. 2012a). Among these, however, we believe only one, or potentially a very small number of closely related viruses are responsible for the production of antibodies detected using the Luminex‐NiVsG assay. The evidence for this is that antibodies detected by this assay correlate well with HeV and NiV neutralizing activity in this, and other bat species (Plowright *et al*. 2008; Breed *et al*. 2010, 2013; Peel *et al*. 2012, 2013), whereas antibodies against the recently identified Cedar virus (CedPV, the third henipavirus), however, are cross‐reactive, but not cross‐neutralizing with HeV and NiV (Marsh *et al*. 2012). This suggests that the virus under study here should have a closer relationship with HeV and NiV than CedPV does. This criterion is only filled by two of the multitude of henipavirus‐like sequence fragments detected in *E. helvum* (Fig. S4). Although our extensive efforts to detect a true African henipavirus have been unsuccessful (Baker *et al*. 2012a,b, 2013), we are confident that the antibody response demonstrated here is directed against one or very few closely related, true henipaviruses.

The cross‐sectional seroprevalence at the outset of the study confirmed that the bat population had been naturally infected with henipaviruses, and the age distribution of seroprevalence was comparable with those found in cross‐sectional field studies (Plowright *et al*. 2008; Breed *et al*. 2011; Peel *et al*. 2012). Here, we set a relatively low threshold for seropositivity to incorporate non‐neutralizing reactivity. Both laboratory and field studies suggest that bats may produce low affinity but broadly reactive antibodies (Bratsch *et al*. 2011; Muller *et al*. 2012; Baker, Schountz & Wang 2013) and that lower thresholds for seropositivity are appropriate (Peel *et al*. 2013). This is also supported by evidence from this study, where very low reactivity samples (i.e. between 2 and 3 mAb m102·4CEs) comprised clearly recognizable immunological trends (e.g. antibody decay and seroconversion). The combined power of the assay and the quantitation method used here enabled the detection of subtle, but significant, changes in antibody concentration in individuals. Notably, although this threshold reliably indicates the presence of antibodies, this is irrespective of their ability to protect individuals from infection, which is discussed further below.

In this study, we were able to demonstrate the existence of maternal antibodies (matAb), long‐suspected from cross‐sectional field studies (Plowright *et al*. 2008; Breed *et al*. 2011; Peel *et al*. 2012) and recently shown for *Pteropus* sp. (Epstein *et al*. 2013). Correlations in both the serostatus and antibody concentrations of dam‐pup pairs indicated that matAbs were present, with pups having slightly higher concentrations than their dams, as in other matAb systems (Lefvert 1998). That neonatal antibodies were the IgG isotype and universally declined in subsequent samplings provided further evidence of their likely maternal origin. Furthermore, these maternal antibodies appeared to offer protection against infection (surrogated in this study by seroconversion). This was shown by the seroconversion of young bats following the decline of maternal antibodies (between 6 and 12 months of age). Bats born seronegative were more likely to have seroconverted by the end of the study, and seroconversion happened at a younger age compared with bats born seropositive. Collectively, this evidence suggests an uncomplicated system, where antibodies in neonates are maternally derived, are protective against infection until their decay, upon which young are susceptible to infection via horizontal transmission.

However, given the conflicting evidence regarding the vertical transmission of henipaviruses (Williamson *et al*. 1999; Halpin *et al*. 2000, 2011), and the possibility of neonatal infection with henipaviruses, it is important to consider evidence contrary to the encompassing statement outlined above. Here, two pups had a serostatus that differed from their dams. Being born in different years with repeatable laboratory results, these likely represent true observations. The seronegative pup born to a seropositive dam was not sampled until 3 months of age (equivalent to 1·7 matAb half‐lives as estimated here) and later was the earliest pup in the study to seroconvert, so it is possible it was born with a low level of matAb that was not observed due to delayed sampling. In the alternate pair (the seropositive pup born to a seronegative dam), the pup had the lowest antibody concentration at birth of any neonate in the study and, following waning of these antibodies, similarly seroconverted comparatively young for its birth cohort (i.e. seropositive bats born in 2010, Table 2, Fig. 5). Given the low antibody concentration in the neonate and that neonatal antibody levels were typically ~35% higher than their dams, it is possible that the dam became seronegative prior to the sampling event. An alternative biological explanation for either of these dam‐pup discrepancies would be allosuckling, which has been reported in bats (Roulin & Heeb 1999). However, owing to the low antibody concentrations involved and subsequent life events, these discrepancies in dam‐pup serostatus most likely arose from observational gaps.

Throughout the course of the study, there was strong evidence of seasonal horizontal transmission among young bats and adult females. Younger, seronegative bats typically seroconverted between 16 and 24 months of age, and these events were clustered in periods corresponding with late pregnancy of adult females (March 2011 and January 2012). These events were coupled in time with increases (both seroconverting and more moderate) in antibody concentrations of adult females. The undulating pattern of seroconversion in adult females with breeding is supported by a similar association of seropositivity with late pregnancy and lactation seen in field studies (Plowright *et al*. 2008; Breed *et al*. 2011). This is probably due to shifts in the immunological response during pregnancy. Typically, late pregnancy is coupled with a depression of cell‐mediated immunity (Boue, Nicolas & Montagnon 1971; Weinberg 1984), and this has been demonstrated for *Myotis* bats (Christe, Arlettaz & Vogel 2000; Baker, Schountz & Wang 2013). Thus, the finding that late pregnancy appears to make adult females susceptible to henipavirus infection might suggest an important role for cell‐mediated immunity in its control outside of these times. Regardless of mechanism, however, the coupling of adult female seroconversions with those of young bats appears to indicate an increase in horizontal transmission during these periods. This seasonal increase in transmission might represent a period of increased zoonotic risk, as infection peaks in juvenile bats have been associated with increased zoonotic spillover of Marburg virus (Amman *et al*. 2012).

Notably, however, these seasonal seroconversions did not affect adult males. The reason for their apparent resistance to these periods of increased transmission is unknown, but it may be that the pregnancy‐related change in immune responses may be the key driving factor of infection in adult bats. Very few seroconversions of adult males occurred throughout the present study and those that did were not coupled in time with pregnancy/lactation. Although explanations regarding the timing of so few, events are somewhat speculative, rather than being associated with pregnancy/lactation as in the adult females and young, seroconversions of adult males occurred in the middle of the year (May 2010 and July 2011), closer to the April – June mating period of *E. helvum* (Mutere 1968), when increased aggression among males and more intimate contact with females is likely. Thus, rather than being associated with increased horizontal transmission during the time of pregnancy/lactation, the few adult male transmissions may have been associated with the mating period.

Here, evidence of active infection in the colony was seen throughout the study period (including in bats born in the facility), but was not first observed until 4 months into the study. The population‐level infection persistence in this small population is consistent with the finding that the small, isolated population of *E. helvum annobonensis* maintains henipavirus infection (Peel *et al*. 2012). The pressing questions then are that of site and mechanism for this population‐level persistence. Where, and in whom, is the virus maintained, and what drives periods of active infection and quiescence? Two potential mechanisms for this population‐level virus persistence are that immunity after a period of infection declines (i.e. SIRS dynamics) or the existence of persistently infected individuals. In the case of the former, the waning‐seroconversion cycle in adult females provides evidence that SIRS dynamics may exist in this system. Furthermore, other studies of henipaviruses in *E. helvum* show a decrease in adult seroprevalence with age in years (Peel 2012), which may also lend itself to such dynamics. In the latter case of persistently infected individuals, theoretical models have shown such individuals would greatly contribute to population‐level persistence of henipaviruses (Plowright *et al*. 2011). Recrudescent henipavirus infection has already been suggested to occur in humans and *Pteropus* bats (Rogers *et al*. 1996; Tan *et al*. 2002; Sohayati *et al*. 2011), and another paramyxovirus (Porcine rubulavirus) is known to persist in the male reproductive tract of pigs for over 4 months (Rivera‐Benitez *et al*. 2013). Also, but speculatively, if vertical infection did occur (although no evidence was found in this study), infected neonates might become immunotolerant to henipaviruses and continue lifelong excretion in a manner similar to Bovine Viral Diarrhoea Virus (Potgieter 1995). In this case, individuals would be born matAb positive, and, following matAb waning, fail to seroconvert on exposure. Indeed, there were some individuals in this study which had failed to seroconvert by ≥4 years of age. To further address questions regarding potential mechanisms of persistence, however, longitudinal molecular virological studies are required (see below).

Another final consideration for understanding viral persistence in a population is the role of population structure and dispersal events in the maintenance of infection. In the current study, naturally occurring population seroprevalences might have been disrupted by the end of the study period, as evidenced by an increase in juvenile seroprevalence to levels comparable to adults. This increase is probably attributable to bats born in 2011 having higher levels of matAb (Table 2, Fig. S2), which were still detectable at 10 months of age. The reasons for the high matAb concentrations in these bats relative to their wild counterparts or pups born in 2010 are unknown, but it is possible that if infection equilibrium previously existed in the wild population, it may be disrupted by the absence of migration, severe reduction in population size and closure to outside infection, as necessitated by the study design. Continued observation of this population over time will help to address these questions.

Detection of virus is an unfortunate gap in this study. In addition to working towards addressing mechanisms of persistence, such data would enable confirmation that observed seroconversions truly are linked with active infection. Although efforts to detect henipavirus RNA in throat swabs taken during this study are ongoing, it was not practically possible to collect urine for molecular analysis. Encouragingly, however, longitudinal molecular studies of wild bat populations show shedding events occurring at life‐history stages that would be predicted by the seroconversions seen during the current study. For example, longitudinal sampling of wild *Pteropus* roosts in Thailand and *Myotis* populations in Germany shows seasonal excretion peaks of henipavirus and coronaviruses, respectively, that are associated with pregnancy and lactation (Gloza‐Rausch *et al*. 2008; Wacharapluesadee *et al*. 2009). Thus, our findings here are potentially generalizable to other systems and may indicate that seasons of late pregnancy/lactation in bat populations might represent periods of increased zoonotic risk.

## Supporting information


**Fig. S1.** which shows laboratory results supporting IgG detection in neonatal samples.
**Fig. S2.** which details information used to infer matAb half‐life.
**Fig. S3.** which tracks individual adult antibody levels over time.
**Fig. S4.** which details the phylogenetic and serological relationships of African henipa‐like viruses.

**Tables S1.** which includes bat details and serum antibody concentrations for all bats and sampling intervals in this study broken down by age.
**Tables S2.** which shows the succinct results for adult bats as in Tables 2 and 3.
