# Supplementary material for: Viral antibody dynamics in a chiropteran host
Source: J Anim Ecol. 2013 Nov 13;83(2):415–28. doi: 10.1111/1365-2656.12153 (PMC4413793; doi:10.1111/1365-2656.12153)
Supplement: Supplementary file 1 — Fig. S1. which shows laboratory results supporting IgG detection in neonatal samples. Fig. S2. which details information used to infer matAb half‐life. Fig. S3. which tracks individual adult antibody levels over time. Fig. S4. which details the phylogenetic and serological relationships of African henipa‐like viruses. [file JANE-83-415-s001.pdf]

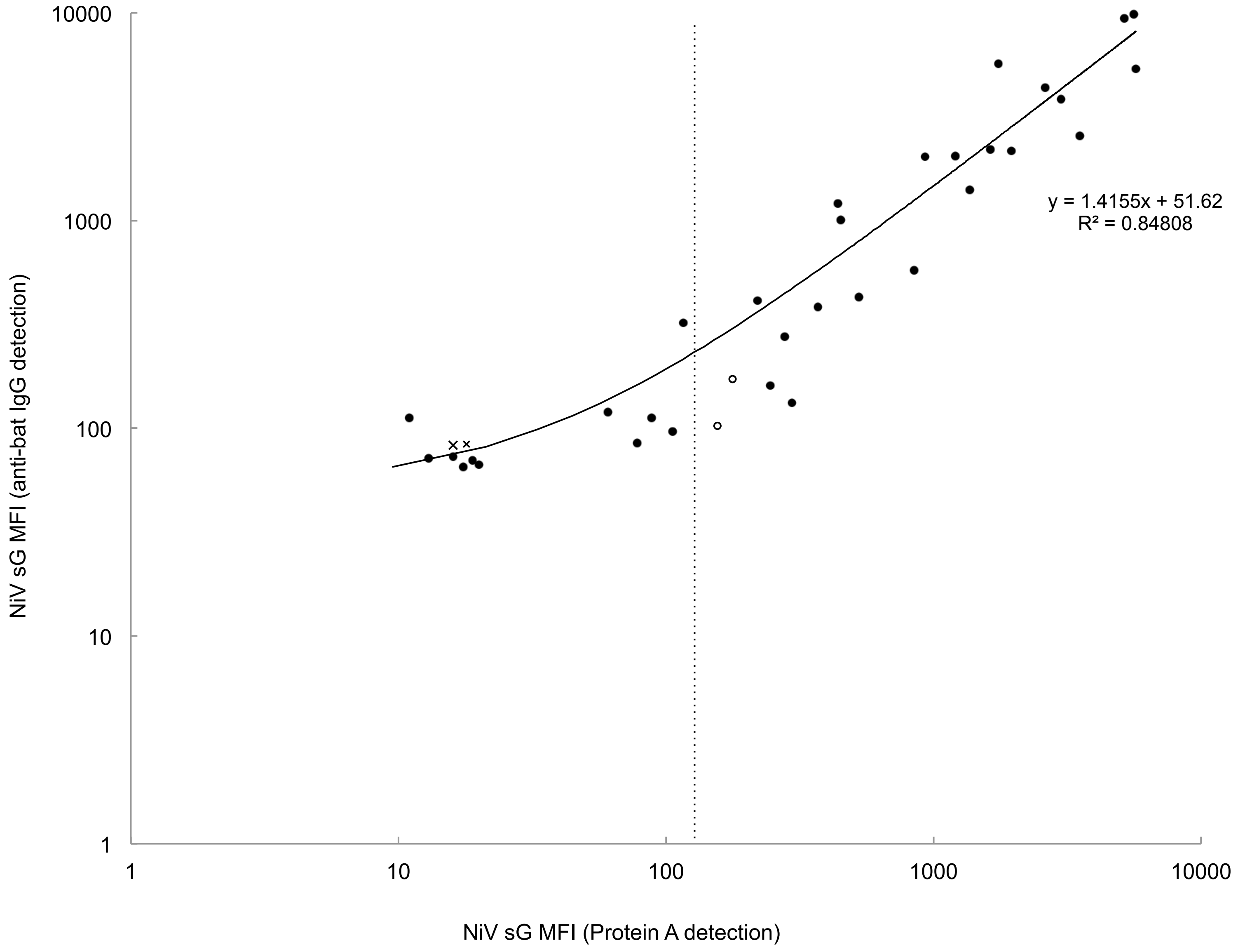

Figure S1. Correlation between Median Fluorescence Intensity generated using an anti-Bat IgG antibody and the less-specific Protein-A conjugate for antibody detection in first-sampling sera from neonatal bats born in captivity in 2010 and 2011. Samples from pups whose serostatus differed from their dam were retested in duplicate and are shown by different markers (the seropositive-pup born to a seronegative-dam is shown by open circles and the seronegative pup born to a seropositive dam is shown by crosses). The vertical dashed line shows the NiVsG MFI (as detected by Protein A) that was used as a threshold for seropositivity in this study.

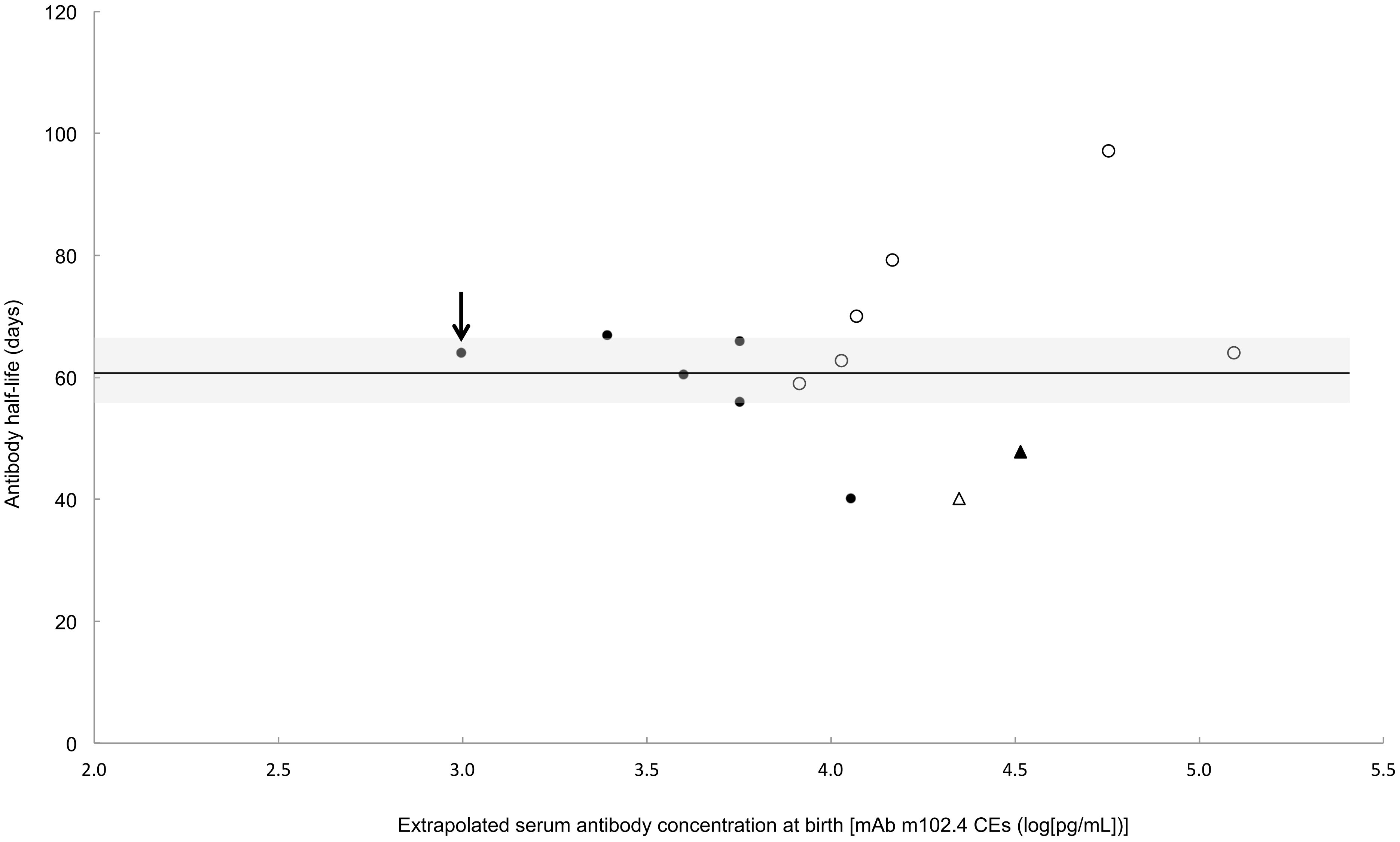

Figure S2. Details of maternal antibody half life approximation. Relationship between antibody half-life in individual captive-born bats and antibody concentration at birth (markers) are shown with solid line representing the antibody half-life calculated using data from all individuals. Grey shading around the lined is the 95% confidence interval. Half-lives were typically calculated from two data points which for bats born in 2010 (closed circles) occurred at 51 and 101 days after birth and for bats born in 2011 (open circles) at 104 and 292 days after birth. Half lives from 2010 calculated from four and five data points are shown as open and closed triangles respectively. The arrow indicates the marker for Bat B153, a seropositive pup born to a seronegative dam.

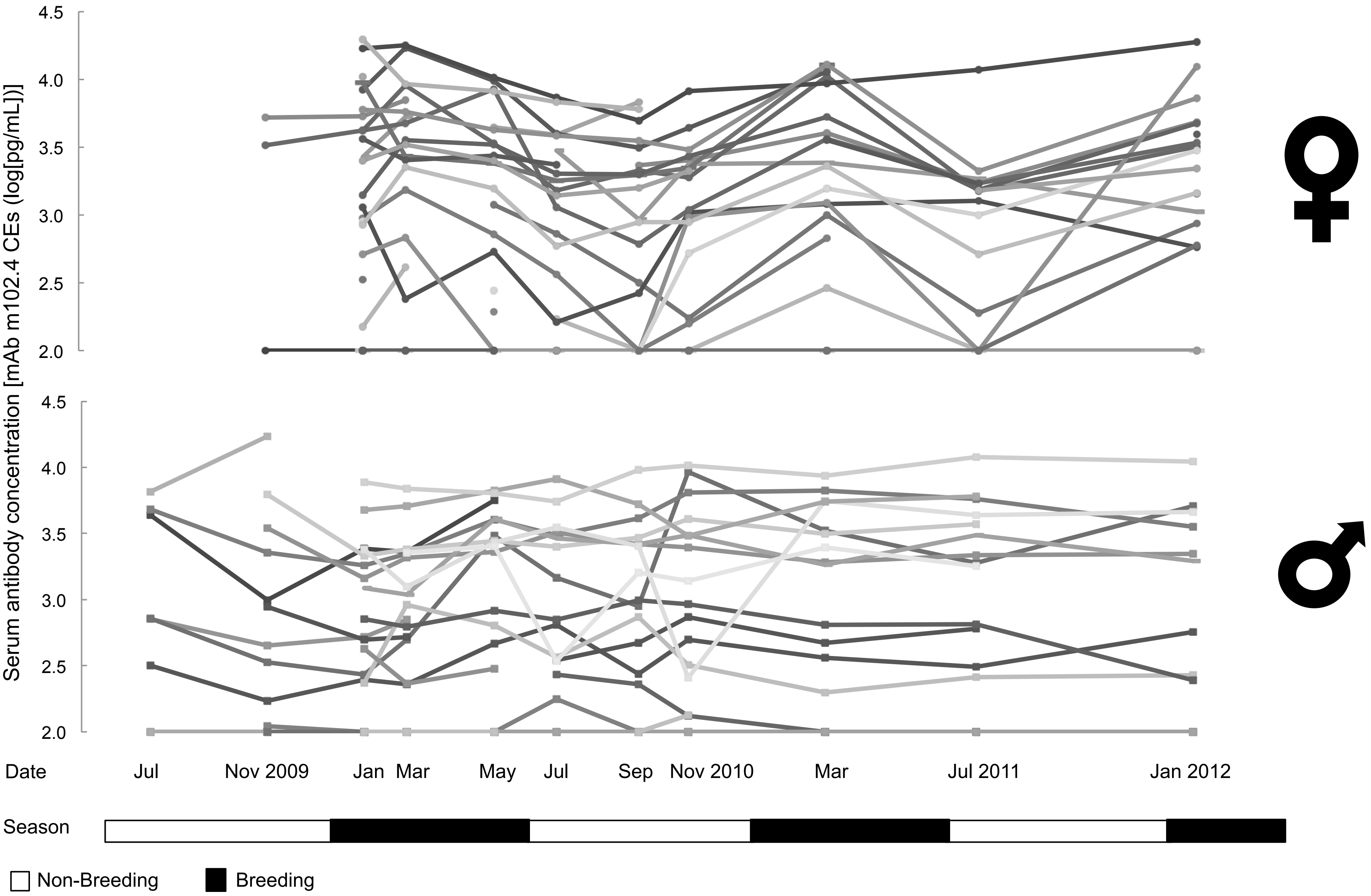

Figure S3. Antibody concentrations over time for individual adult bats (markers represent measured values, lines present to demonstrate trends). Females are shown on the graph above and males below. The sampling interval and an indication of breeding status are shown below.

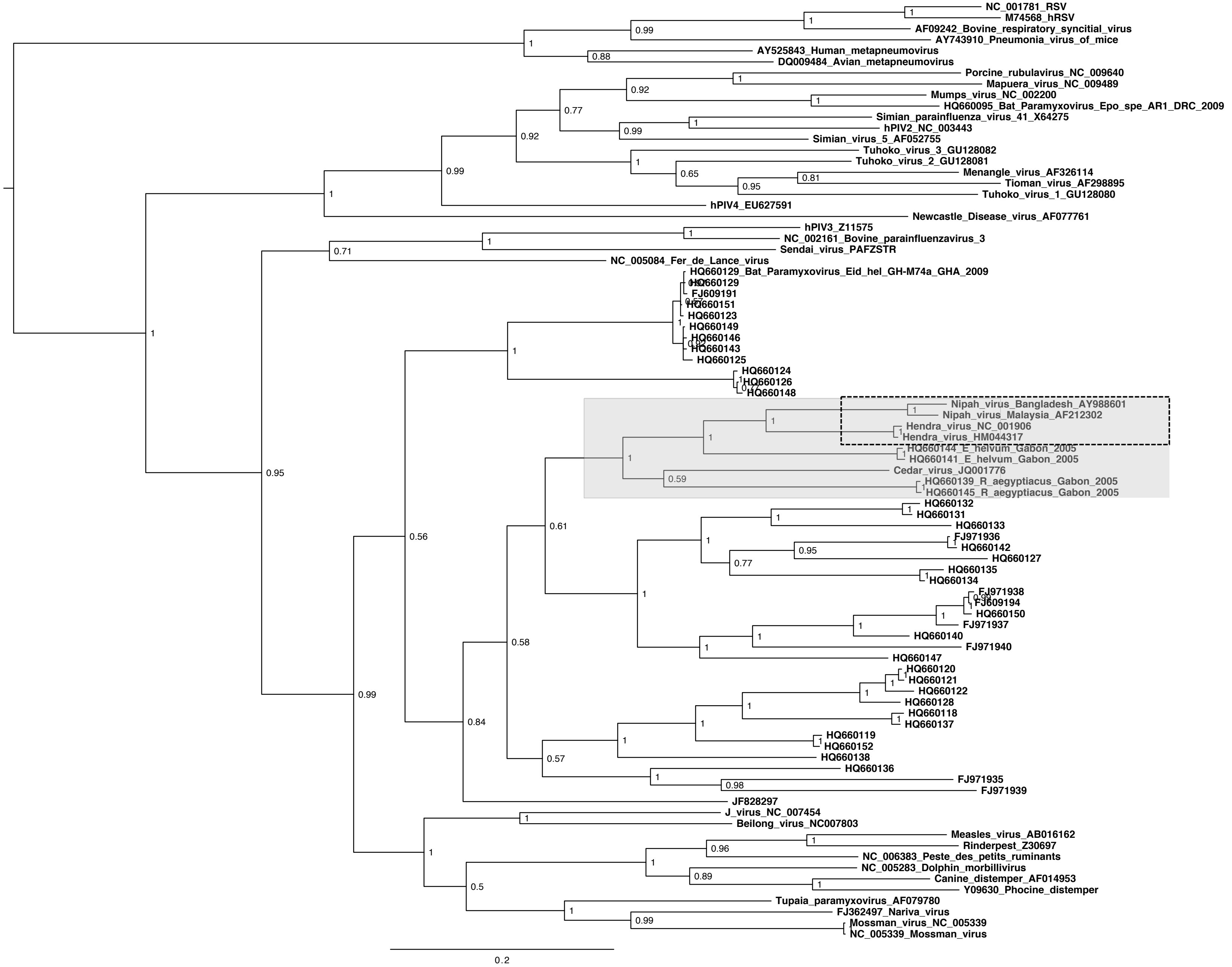

Figure S4. Phylogenetic tree inferred though Bayesian analysis (as in Baker et al 2012b) of a 559 nucleotide alignment of *Paramyxovirinae* polymerase genes and available gene fragments of henipa-like viruses described in Drexler et al (2012). Node values show posterior probability values for each clade. The clade containing serologically cross-reactive henipaviruses (Hendra, Nipah and Cedar viruses) is highlighted in grey, with the sub-clade containing viruses whose anti sera cross-neutralise (Hendra and Nipah viruses only) being highlighted further by enclosure in the dashed lines.
