# Supplementary material for: Viral antibody dynamics in a chiropteran host
Source: J Anim Ecol. 2013 Nov 13;83(2):415–28. doi: 10.1111/1365-2656.12153 (PMC4413793; doi:10.1111/1365-2656.12153)
Supplement: Supplementary file 2 — Tables S1. which includes bat details and serum antibody concentrations for all bats and sampling intervals in this study broken down by age. Tables S2. which shows the succinct results for adult bats as in Tables 2 and 3. [file JANE-83-415-s002.pdf]

| | | Age at entry | Gender | Mortality | | Pregnancy status | | | Dams | mAb102.4 (log[pg/mL]) Concentration Equivalents | | | | | | | | | | |
|---|---|---|---|---|---|---|---|---|---|---|---|---|---|---|---|---|---|---|---|---|
| | | | | | | | | | | Jul-09 | Nov-09 | Jan-10 | Mar-10 | May-10 | Jul-10 | Sep-10 | Nov-10 | Mar-11 | Jul-11 | Jan-12 |
| Entry Cohort | Bat ID * | (approx. months) | | Exit means | Before resampling | Mar-10 | Mar-11 | Jan-12 | (BICs only) | 28/07/2009 | 06/11/2009 | 29/01/2010 | 07/03/2010 | 22/05/2010 | 15/07/2010 | 24/09/2010 | 06/11/2010 | 05/03/2011 | 14/07/2011 | 18/01/2012 |
| 1 | A191 | A | M | Missing | No | | | | | 3.8 | 4.2 | | | | | | | | | |
| 1 | A192 | A | M | | | | | | | 2.9 | 2.5 | 2.4 | 2.7 | 3.5 | 3.2 | 2.9 | 4.0 | 3.5 | 3.3 | 3.7 |
| 1 | A189 | A | M | | | | | | | <2 | <2 | <2 | | | 2.4 | 2.4 | 2.1 | <2 | <2 | <2 |
| 1 | A197 | A | M | | | | | | | <2 | <2 | <2 | <2 | <2 | <2 | <2 | <2 | <2 | <2 | <2 |
| 1 | P100 | A | M | Missing | Yes | | | | | 3.2 | | | | | | | | | | |
| 1 | P75 | A | M | Missing | Yes | | | | | <2 | | | | | | | | | | |
| 1 | P89 | A | M | Missing | Yes | | | | | <2 | | | | | | | | | | |
| 1 | A190 | A | M | Dead | No | | | | | 3.6 | 3.0 | 3.4 | 3.4 | 3.7 | | | | | | |
| 1 | A198 | A | M | Dead | No | | | | | 2.9 | 2.7 | 2.7 | 2.9 | | | | | | | |
| 1 | A195 | A | M | | | | | | | 2.5 | 2.2 | 2.4 | 2.4 | 2.7 | 2.8 | 2.4 | 2.7 | 2.6 | 2.5 | 2.8 |
| 1 | A193 | A | M | | | | | | | 3.7 | 3.4 | 3.3 | 3.3 | 3.6 | 3.5 | 3.6 | 3.8 | 3.8 | 3.8 | 3.6 |
| 1 | A196 | SIM (15m) | M | | | | | | | 2.9 | 3.5 | 3.5 | | 3.2 | 3.3 | 3.2 | 3.5 | 3.4 | 3.4 | 3.6 |
| 2 | A111 | A | F | | | KP | P | N | | | <2 | <2 | <2 | <2 | <2 | <2 | <2 | <2 | <2 | <2 |
| 2 | A106 | A | F | | | KP | N | N | | | 3.5 | 3.6 | 4.0 | 3.5 | 3.2 | 3.3 | 3.3 | 4.0 | 3.2 | 3.5 |
| 2 | A102 | A | F | | | P | P | P | | | 3.7 | 3.7 | 3.8 | | | 3.4 | 3.4 | 3.6 | 3.2 | 3.7 |
| 2 | A105 | A | M | | | | | | | | 2.0 | <2 | <2 | <2 | 2.2 | <2 | <2 | <2 | <2 | <2 |
| 2 | A113 | A | M | | | | | | | | <2 | <2 | <2 | <2 | <2 | <2 | <2 | <2 | <2 | |
| 2 | A092/A103 | A | M | | | | | | | | 3.8 | 3.3 | 3.4 | 3.4 | 3.4 | 3.5 | 3.6 | 3.5 | 3.6 | |
| 2 | A110 | A | M | | | | | | | | 3.5 | 3.2 | 3.3 | 3.4 | 3.5 | 3.4 | 3.4 | 3.3 | 3.3 | 3.3 |
| 2 | A107/A090 | A | M | | | | | | | | 2.9 | 2.7 | 2.7 | | 2.5 | 2.7 | 2.9 | 2.7 | 2.8 | |
| 2 | A101 | JUV (7m) | M | | | | | | | | <2 | <2 | <2 | <2 | 2.7 | 3.0 | 3.0 | | 3.1 | |
| 2 | A112 | JUV (7m) | M | | | | | | | | 3.4 | 3.6 | <2 | <2 | <2 | | <2 | | <2 | <2 |
| 2 | A109 | SIM (19m) | F | Missing | No | A | ND | NA | | | 3.0 | | 3.2 | 3.3 | 3.1 | 3.0 | 3.0 | | | |
| 2 | A108 | SIM (19m) | F | Dead | No | N | NA | NA | | | 4.4 | 4.5 | 4.7 | 4.3 | 4.5 | 4.7 | | | | |
| 2 | A104 | SIM (19m) | F | Missing | Yes | NA | NA | NA | | | 3.4 | | | | | | | | | |
| 3 | A156 | A | F | Dead | No | A | NA | NA | | | | 4.3 | 4.0 | 3.9 | 3.8 | 3.8 | | | | |
| 3 | A162 | A | F | | | A | P | N | | | | <2 | <2 | <2 | | <2 | <2 | <2 | 1.6 | 2.8 |
| 3 | A167 | A | F | Dead | No | A | NA | NA | | | | <2 | <2 | <2 | | | | | | |
| 3 | A141 | A | F | | | A | P | N | | | | 3.4 | 3.7 | | 3.5 | 3.0 | 3.4 | 3.4 | 3.3 | 3.0 |
| 3 | A163 | A | F | | | A | P | P | | | | 2.9 | 3.4 | 3.2 | 2.8 | 2.9 | 2.9 | 3.4 | 2.7 | 3.2 |
| 3 | A158 | A | F | | | A | P | ND | | | | 3.6 | 3.4 | 3.4 | 3.4 | | | 3.6 | 3.2 | |
| 3 | A151 | A | F | | | A | KP | P | | | | 3.1 | 2.4 | 2.7 | 2.2 | 2.4 | 3.0 | 3.1 | 3.1 | 2.8 |
| 3 | A149 | A | F | | | A | P | ND | | | | <2 | <2 | <2 | <2 | <2 | <2 | <2 | <2 | |
| 3 | A160 | A | F | | | A | N | N | | | | 2.9 | | 2.4 | | <2 | 2.7 | 3.2 | 3.0 | 3.5 |
| 3 | A142 | A | F | | | A | P | ND | | | | 4.0 | 3.4 | 3.4 | 3.3 | 3.3 | 3.3 | 4.1 | | |
| 3 | A164 | A | F | | | A | P | P | | | | 3.8 | 3.8 | 3.6 | 3.6 | 3.5 | 3.5 | 4.1 | 3.3 | 3.9 |
| 3 | A143 | A | F | | | A | N | N | | | | 4.2 | 4.3 | 4.0 | 3.9 | 3.7 | 3.9 | 4.0 | 4.1 | 4.3 |
| 3 | A153 | A | F | | | KP | P | P | | | | 2.7 | 2.8 | <2 | <2 | <2 | 3.0 | 3.1 | <2 | 4.1 |
| 3 | A145 | A | F | Dead | No | KP | NA | NA | | | | <2 | | 2.3 | | | | | | |
| 3 | A154/A188 | A | F | | | KP | P | N | | | | <2 | <2 | <2 | <2 | <2 | <2 | <2 | <2 | <2 |
| 3 | A157 | A | F | | | KP | ND | P | | | | <2 | <2 | <2 | <2 | <2 | <2 | | <2 | <2 |
| 3 | A120 | A | F | | | KP | P | P | | | | 2.5 | | 3.1 | 2.9 | 2.5 | 2.2 | 3.0 | 2.3 | 2.9 |
| 3 | A147 | A | F | | | KP | P | P | | | | 3.1 | 3.6 | 3.5 | 3.3 | 3.3 | 3.4 | 3.7 | 3.2 | 3.7 |
| 3 | A150 | A | F | | | KP | P | P | | | | 3.0 | 3.2 | 2.9 | 2.6 | <2 | 2.2 | 2.8 | | 3.2 |
| 3 | A132 | A | F | | | KP | P | N | | | | 3.9 | 4.2 | 4.0 | 3.6 | 3.5 | 3.6 | 4.1 | | 3.6 |
| 3 | A159 | A | F | | | N | N | N | | | | <2 | | <2 | <2 | <2 | <2 | <2 | <2 | <2 |
| 3 | A168/A094/A091 | A | F | | | N | N | P | | | | 3.4 | 3.5 | 3.4 | 3.1 | 3.2 | 3.3 | | 3.2 | 3.3 |
| 3 | A125/A096/A095 | A | F | Dead | No | N | NA | NA | | | | 4.0 | | 3.6 | 3.6 | 3.8 | | | | |
| 3 | A127 | A | F | Dead | Yes | NA | NA | NA | | | | 4.5 | | | | | | | | |
| 3 | A138 | A | F | Dead | Yes | NA | NA | NA | | | | 3.0 | | | | | | | | |
| 3 | A139 | A | F | Dead | Yes | NA | NA | NA | | | | 2.8 | | | | | | | | |
| 3 | A140 | A | F | Missing | Yes | NA | NA | NA | | | | 2.9 | | | | | | | | |
| 3 | A146 | A | F | | | P | P | ND | | | | 2.2 | 2.6 | | 2.2 | <2 | <2 | 2.5 | <2 | |
| 3 | A155 | A | F | | | P | N | P | | | | 3.6 | 3.7 | 3.9 | 3.1 | 2.8 | 3.0 | 3.6 | 3.2 | 3.5 |
| 3 | A114 | A | M | Missing | No | | | | | | | <2 | <2 | | | <2 | <2 | | | |
| 3 | A123 | A | M | | | | | | | | | 2.4 | 3.0 | 2.8 | 2.6 | 2.9 | 2.5 | 2.3 | 2.4 | 2.4 |
| 3 | A135 | A | M | | | | | | | | | <2 | <2 | <2 | <2 | <2 | <2 | <2 | <2 | <2 |
| 3 | A136 | A | M | Missing | No | | | | | | | <2 | <2 | <2 | | <2 | 2.1 | | | |
| 3 | A133 | A | M | Dead | Yes | | | | | | | 3.7 | | | | | | | | |
| 3 | A119 | A | M | | | | | | | | | 3.7 | 3.7 | 3.8 | 3.9 | 3.7 | 3.5 | 3.7 | 3.8 | |
| 3 | A131 | A | M | | | | | | | | | 3.1 | 3.0 | 3.6 | 3.5 | 3.4 | 3.5 | 3.3 | 3.5 | 3.3 |
| 3 | A117 | A | M | Missing | No | | | | | | | 2.6 | 2.4 | 2.5 | | | | | | |
| 3 | A118 | A | M | | | | | | | | | 2.9 | 2.8 | 2.9 | 2.8 | 3.0 | 3.0 | 2.8 | 2.8 | 2.4 |
| 3 | A124 | A | M | | | | | | | | | 3.4 | 3.1 | 3.4 | 3.5 | 3.4 | 2.4 | 3.7 | 3.6 | 3.7 |
| 3 | A128 | A | M | | | | | | | | | | 3.4 | 3.4 | 2.5 | 3.2 | 3.1 | 3.4 | 3.3 | |
| 3 | A121 | A | M | | | | | | | | | 3.9 | 3.8 | 3.8 | 3.7 | 4.0 | 4.0 | 3.9 | 4.1 | 4.0 |
| 3 | A152 | JUV (9m) | F | | | A | N | ND | | | | <2 | <2 | <2 | <2 | <2 | <2 | 4.5 | 3.9 | 4.4 |
| 3 | A166/A093/A089 | JUV (9m) | F | | | NA | N | ND | | | | <2 | <2 | <2 | <2 | <2 | <2 | 3.8 | | |
| 3 | A098 | JUV (9m) | F | | | NA | KP | N | | | | <2 | <2 | <2 | | 2.9 | 3.1 | 3.9 | 3.8 | 4.1 |
| 3 | A099 | JUV (9m) | F | | | NA | P | N | | | | <2 | <2 | <2 | | <2 | <2 | <2 | <2 | 4.4 |
| 3 | A115 | JUV (9m) | M | | | | | | | | | <2 | <2 | <2 | <2 | <2 | 3.1 | | 4.2 | 4.4 |
| 3 | A130 | JUV (9m) | M | | | | | | | | | <2 | <2 | <2 | <2 | <2 | 2.5 | 3.5 | 3.7 | |
| 3 | A148 | SIM (21m) | F | | | A | ND | N | | | | 4.7 | 4.6 | 4.1 | 4.3 | 4.3 | | | 3.8 | 4.5 |
| 3 | A097 | SIM (21m) | F | | | N | N | N | | | | <2 | <2 | <2 | <2 | <2 | <2 | 2.9 | 3.7 | 3.8 |
| 3 | A144 | SIM (21m) | F | | | P | KP | N | | | | 2.8 | <2 | <2 | <2 | <2 | <2 | 3.3 | 3.1 | 3.2 |
| 3 | A134 | SIM (21m) | M | | | | | | | | | <2 | <2 | | | <2 | <2 | <2 | <2 | <2 |
| 3 | A122 | SIM (21m) | M | | | | | | | | | 3.2 | 3.0 | 3.7 | 3.5 | 3.3 | 3.5 | 3.4 | 3.4 | |
| 3 | A126 | SIM (21m) | M | | | | | | | | | 2.6 | 2.5 | 2.9 | | 3.0 | 3.0 | 3.0 | 2.8 | 2.9 |
| 4 | B188 | BIC | F | | | | | | A188 | | | | | <2 | <2 | <2 | <2 | <2 | 3.0 | |
| 4 | B111 | BIC | M | | | | | | A111 | | | | | <2 | | <2 | <2 | <2 | | |
| 4 | B153 | BIC | F | | | | | | A153 | | | | | 2.8 | 2.5 | <2 | <2 | <2 | <2 | 3.1 |
| 4 | B120 | BIC | F | | | | | | A120 | | | | | 3.2 | 2.9 | <2 | <2 | <2 | <2 | 2.2 |
| 4 | BJ1 | BIC | F | | | | | | ND | | | | | | <2 | <2 | <2 | <2 | <2 | 3.0 |
| 4 | B157/A083 | BIC | M | | | | | | A157 | | | | | <2 | <2 | <2 | <2 | <2 | | 2.7 |
| 4 | B145 | BIC | M | Missing | Yes | | | | A145 | | | | | 2.0 | | | | | | |
| 4 | B147 | BIC | F | Dead | No | | | | A147 | | | | | 3.7 | 3.3 | | | | | |
| 4 | B150 | BIC | F | | | | | | A150 | | | | | 3.4 | | <2 | <2 | <2 | | <2 |
| 4 | B106/BJ6 | BIC | F | | | | | | A106 | | | | | 4.1 | 3.4 | | 2.8 | | | 2.7 |
| 4 | B132 | BIC | M | | | | | | A132 | | | | | 4.3 | 3.7 | 3.5 | 3.0 | 2.5 | <2 | <2 |
| 5 | A0004 | BIC | F | | | | | | ND | | | | | | | | | | 3.4 | 2.4 |
| 5 | A081 | BIC | M | | | | | | ND | | | | | | | | | | 3.5 | 2.6 |
| 5 | 7158 | BIC | M | | | | | | ND | | | | | | | | | | 3.6 | 2.8 |
| 5 | 9186 | BIC | ND | | | | | | A144 | | | | | | | | | | <2 | 2.9 |
| 5 | A001 | BIC | F | | | | | | ND | | | | | | | | | | 3.8 | 3.1 |
| 5 | 3940 | BIC | F | | | | | | ND | | | | | | | | | | 4.6 | 3.7 |
| 5 | 6544 | BIC | F | | | | | | ND | | | | | | | | | | 4.4 | 3.9 |
| 5 | 7034 | BIC | F | | | | | | ND | | | | | | | | | | <2 | <2 |
| 5 | A175 | BIC | F | | | | | | ND | | | | | | | | | | <2 | <2 |
| 5 | A075 | BIC | F | | | | | | ND | | | | | | | | | | 2.2 | <2 |
| 5 | 3428 | BIC | M | | | | | | ND | | | | | | | | | | 3.1 | <2 |
| 5 | 9308 | BIC | F | | | | | | ND | | | | | | | | | | 3.7 | |
| 5 | 829 | BIC | ND | | | | | | A151 | | | | | | | | | | 3.6 | |
| 5 | A002 | BIC | ND | | | | | | ND | | | | | | | | | | 3.9 | |
| 5 | A003 | BIC | ND | | | | | | ND | | | | | | | | | | 4.0 | |
| 5 | 349 | BIC | ND | | | | | | ND | | | | | | | | | | 2.8 | |
| 5 | 162 | BIC | ND | | | | | | ND | | | | | | | | | | <2 | |
| 5 | 231 | BIC | ND | | | | | | A098 | | | | | | | | | | 4.5 | |
| 5 | 569 | BIC | ND | | | | | | ND | | | | | | | | | | 4.1 | |
| 5 | 647 | BIC | ND | | | | | | ND | | | | | | | | | | 2.7 | |
| 5 | 764 | BIC | ND | | | | | | ND | | | | | | | | | | 2.7 | |
| 5 | 332 | BIC | ND | | | | | | ND | | | | | | | | | | <2 | |

Notations

| | | | | | | | | |
|---|---|---|---|---|---|---|---|---|
| BIC | Born in Captivity | M | Male | P | Pregnant | (checkered) Sample not taken | (blue) Non-breeding |
| A | Adult | F | Female | KP | Known pup | (black) Bat has exited study | (yellow) Pregnancy/lactation period |
| JUV | Juvenile | NA | Not Applicable | N | Non-pregnant | | |
| SIM | Sexually Immature | ND | Not Determined | A | Aborted pregnancy | | |

**Mortality**

Missing: Missing ≥ 3 sampling events

Dead: Known mortality

* Multiple collar names are a single bat with multiple bats (tracked through PIT tag number)

| | | | | mAb102.4 CEs (log[pg/mL]) by sampling date | | | | | | | | | | |
|---|---|---|---|---|---|---|---|---|---|---|---|---|---|---|
| | | | | Days since last bleed | 101 | 84 | 37 | 76 | 54 | 71 | 43 | 119 | 131 | 188 |
| BatID | Pregnancy status | | | | | | | | | | | | | |
| Females | Mar-10 | Mar-11 | Jan-12 | **28/07/2009** | **06/11/2009** | **29/01/2010** | **07/03/2010** | **22/05/2010** | **15/07/2010** | **24/09/2010** | **06/11/2010** | **05/03/2011** | **14/07/2011** | **18/01/2012** |
| A102 | P | P | P | | 3.7 | 3.7 | 3.8 | | | 3.4 | 3.4 | 3.6 | 3.2 | 3.7 |
| A106 | KP | N | N | | 3.5 | 3.6 | 4.0 | 3.5 | 3.2 | 3.3 | 3.3 | **4.0** | 3.2 | 3.5 |
| A111 | KP | P | N | | <2 | <2 | <2 | <2 | <2 | <2 | <2 | <2 | <2 | <2 |
| A120 | KP | P | P | | | 2.5 | | 3.1 | 2.9 | 2.5 | 2.2 | **3.0** | 2.3 | **2.9** |
| A125/A096/A095 | N | NA | NA | | | 4.0 | | 3.6 | 3.6 | 3.8 | | | | |
| A132 | KP | P | N | | | 3.9 | 4.2 | 4.0 | 3.6 | 3.5 | 3.6 | 4.1 | | 3.6 |
| A141 | A | P | N | | | 3.4 | 3.7 | | 3.5 | 3.0 | 3.4 | 3.4 | 3.3 | 3.0 |
| A142 | A | P | ND | | | 4.0 | 3.4 | 3.4 | 3.3 | 3.3 | 3.3 | **4.1** | | |
| A143 | A | N | N | | | 4.2 | 4.3 | 4.0 | 3.9 | 3.7 | 3.9 | 4.0 | 4.1 | 4.3 |
| A145 | KP | NA | NA | | | <2 | | 2.3 | | | | | | |
| A146 | P | P | ND | | | 2.2 | 2.6 | | 2.2 | <2 | <2 | 2.5 | <2 | |
| A147 | KP | P | P | | | 3.1 | 3.6 | 3.5 | 3.3 | 3.3 | 3.4 | 3.7 | 3.2 | 3.7 |
| A149 | A | P | ND | | | <2 | <2 | <2 | <2 | <2 | <2 | <2 | <2 | |
| A150 | KP | P | P | | | 3.0 | 3.2 | 2.9 | 2.6 | <2 | 2.2 | **2.8** | | 3.2 |
| A151 | A | KP | P | | | 3.1 | 2.4 | 2.7 | 2.2 | 2.4 | 3.0 | 3.1 | 3.1 | 2.8 |
| A153 | KP | P | P | | | 2.7 | 2.8 | <2 | <2 | <2 | **3.0** | 3.1 | <2 | 4.1 |
| A154/A188 | KP | P | N | | | <2 | <2 | <2 | <2 | <2 | <2 | <2 | <2 | <2 |
| A155 | P | N | P | | | 3.6 | 3.7 | 3.9 | 3.1 | 2.8 | 3.0 | 3.6 | 3.2 | 3.5 |
| A156 | A | NA | NA | | | 4.3 | 4.0 | 3.9 | 3.8 | 3.8 | | | | |
| A157 | KP | ND | P | | | <2 | <2 | <2 | <2 | <2 | <2 | | <2 | <2 |
| A158 | A | P | ND | | | 3.6 | 3.4 | 3.4 | 3.4 | | | 3.6 | 3.2 | |
| A159 | N | N | N | | | <2 | | <2 | <2 | <2 | <2 | <2 | <2 | <2 |
| A160 | A | N | N | | | 2.9 | | 2.4 | | <2 | 2.7 | 3.2 | 3.0 | 3.5 |
| A162 | A | P | N | | | <2 | <2 | <2 | | <2 | <2 | <2 | <2 | **2.8** |
| A163 | A | P | P | | | 2.9 | 3.4 | 3.2 | 2.8 | 2.9 | 2.9 | 3.4 | 2.7 | 3.2 |
| A164 | A | P | P | | | 3.8 | 3.8 | 3.6 | 3.6 | 3.5 | 3.5 | **4.1** | 3.3 | 3.9 |
| A167 | A | NA | NA | | | <2 | <2 | <2 | | | | | | |
| A168/A094/A091 | N | N | P | | | 3.4 | 3.5 | 3.4 | 3.1 | 3.2 | 3.3 | | 3.2 | 3.3 |
| Males | | | | | | | | | | | | | | |
| A195 | | | | 2.5 | 2.2 | 2.4 | 2.4 | 2.7 | 2.8 | 2.4 | 2.7 | 2.6 | 2.5 | 2.8 |
| A198 | | | | 2.9 | 2.7 | 2.7 | 2.9 | | | | | | | |
| A192 | | | | 2.9 | 2.5 | 2.4 | 2.7 | **3.5** | 3.2 | 2.9 | **4.0** | 3.5 | 3.3 | 3.7 |
| A190 | | | | 3.6 | 3.0 | 3.4 | 3.4 | 3.7 | | | | | | |
| A193 | | | | 3.7 | 3.4 | 3.3 | 3.3 | 3.6 | 3.5 | 3.6 | 3.8 | 3.8 | 3.8 | 3.6 |
| A191 | | | | 3.8 | 4.2 | | | | | | | | | |
| A189 | | | | <2 | <2 | <2 | | | 2.4 | 2.4 | 2.1 | <2 | <2 | <2 |
| A197 | | | | <2 | <2 | <2 | <2 | <2 | <2 | <2 | <2 | <2 | <2 | <2 |
| A105 | | | | | 2.0 | <2 | <2 | <2 | 2.2 | <2 | <2 | <2 | <2 | <2 |
| A107/A090 | | | | | 2.9 | 2.7 | 2.7 | | 2.5 | 2.7 | 2.9 | 2.7 | 2.8 | |
| A110 | | | | | 3.5 | 3.2 | 3.3 | 3.4 | 3.5 | 3.4 | 3.4 | 3.3 | 3.3 | 3.3 |
| A092/A103 | | | | | 3.8 | 3.3 | 3.4 | 3.4 | 3.4 | 3.5 | 3.6 | 3.5 | 3.6 | |
| A113 | | | | | <2 | <2 | <2 | <2 | <2 | <2 | <2 | <2 | <2 | |
| A123 | | | | | | 2.4 | 3.0 | 2.8 | 2.6 | 2.9 | 2.5 | 2.3 | 2.4 | 2.4 |
| A117 | | | | | | 2.6 | 2.4 | 2.5 | | | | | | |
| A118 | | | | | | 2.9 | 2.8 | 2.9 | 2.8 | 3.0 | 3.0 | 2.8 | 2.8 | 2.4 |
| A131 | | | | | | 3.1 | 3.0 | 3.6 | 3.5 | 3.4 | 3.5 | 3.3 | 3.5 | 3.3 |
| A124 | | | | | | 3.4 | 3.1 | 3.4 | 3.5 | 3.4 | 2.4 | **3.7** | 3.6 | 3.7 |
| A119 | | | | | | 3.7 | 3.7 | 3.8 | 3.9 | 3.7 | 3.5 | 3.7 | 3.8 | |
| A121 | | | | | | 3.9 | 3.8 | 3.8 | 3.7 | 4.0 | 4.0 | 3.9 | 4.1 | 4.0 |
| A114 | | | | | | <2 | <2 | | | <2 | <2 | | | |
| A135 | | | | | | <2 | <2 | <2 | <2 | <2 | <2 | <2 | <2 | <2 |
| A136 | | | | | | <2 | <2 | <2 | | <2 | 2.1 | | | |
| A128 | | | | | | | 3.4 | 3.4 | 2.5 | **3.2** | 3.1 | 3.4 | 3.3 | |

* Multiple collar names are a single bat with multiple bats (tracked through PIT tag number)

Sample not taken

P Pregnant

KP Known pup

N Non-pregnant

A Aborted pregnancy
